# A Simplified Method for the Aspiration of Bone Marrow from Patients Undergoing Hip and Knee Joint Replacement for Isolating Mesenchymal Stem Cells and* In Vitro* Chondrogenesis

**DOI:** 10.1155/2016/3152065

**Published:** 2016-02-11

**Authors:** Subhash C. Juneja, Sowmya Viswanathan, Milan Ganguly, Christian Veillette

**Affiliations:** ^1^Arthritis Program, Orthopaedic Surgery, Toronto Western Hospital, 399 Bathurst Street, Toronto, ON, Canada M5T 2S8; ^2^Institute of Biomaterials and Biomedical Engineering, University of Toronto, 164 College Street, Toronto, ON, Canada M5S 3G9; ^3^Krembil Research Institute, University Health Network, 399 Bathurst Street, Toronto, ON, Canada M5T 2S8; ^4^STTARR Innovation Centre, Princess Margaret Cancer Centre, University Health Network, 101 College Street, Toronto, ON, Canada M5G 1L7

## Abstract

The procedure for aspiration of bone marrow from the femur of patients undergoing total knee arthroplasty (TKA) or total hip arthroplasty (THA) may vary from an OR (operating room) to OR based on the surgeon's skill and may lead to varied extent of clotting of the marrow and this, in turn, presents difficulty in the isolation of mesenchymal stem cells (MSCs) from such clotted bone marrow. We present a simple detailed protocol for aspirating bone marrow from such patients, isolation, and characterization of MSCs from the aspirated bone marrow specimens and show that the bone marrow presented no clotting or exhibited minimal clotting. This represents an economical source and convenient source of MSCs from bone marrow for use in regenerative medicine. Also, we presented the detailed protocol and showed that the MSCs derived from such bone marrow specimens exhibited MSCs characteristics and generated micromass cartilages, the recipe for regenerative medicine for osteoarthritis. The protocols we presented can be used as standard operating procedures (SOPs) by researchers and clinicians.

## 1. Introduction

The posterior iliac crest is a readily accessible site for the bone marrow aspiration that is safe and psychologically less traumatic and affords representative samples of bone marrow similar to that obtained from the sternum, the vertebral spine, and the anterior iliac crest [1]. In general, for biopsy and clinical diagnosis, clinicians collect bone marrow from posterior and anterior iliac crest and lumbar pedicle. For osteoarthritis research and for developing bioengineering tools for repairing OA or use in other diseases, bone marrow can be aspirated from the femur of patients undergoing TKA or THA and can be an alternative and economical source of mesenchymal stem cells (MSCs). There are reports of researchers using bone marrow aspirated from patients for research though protocol details have never been published [2, 3]. Bone marrow aspiration procedure from patients may vary from surgeon to surgeon. In the beginning, surgeons in our OR tried to aspirate bone marrow from patient's femur with a pipette and that resulted in clotting of bone marrow. By introducing this simple procedure, the bone marrow is aspirated directly into the specimen trap and gets mixed with anticoagulant instantly under the influence of vacuum resulting in minimum or no clotting of bone marrow. Second, we present a simple lab procedure for isolating MSCs and detail protocol for generating micromass cartilages that can be used for osteoarthritis research and repair. As far as we know, this is the first detailed report for a simplified procedure for aspirating bone marrow.

## 2. Materials and Methods

### 2.1. Aspiration of Bone Marrow from Patients Undergoing Total Knee or Hip Arthroplasty (TKA or THA)

**Figure 1:**
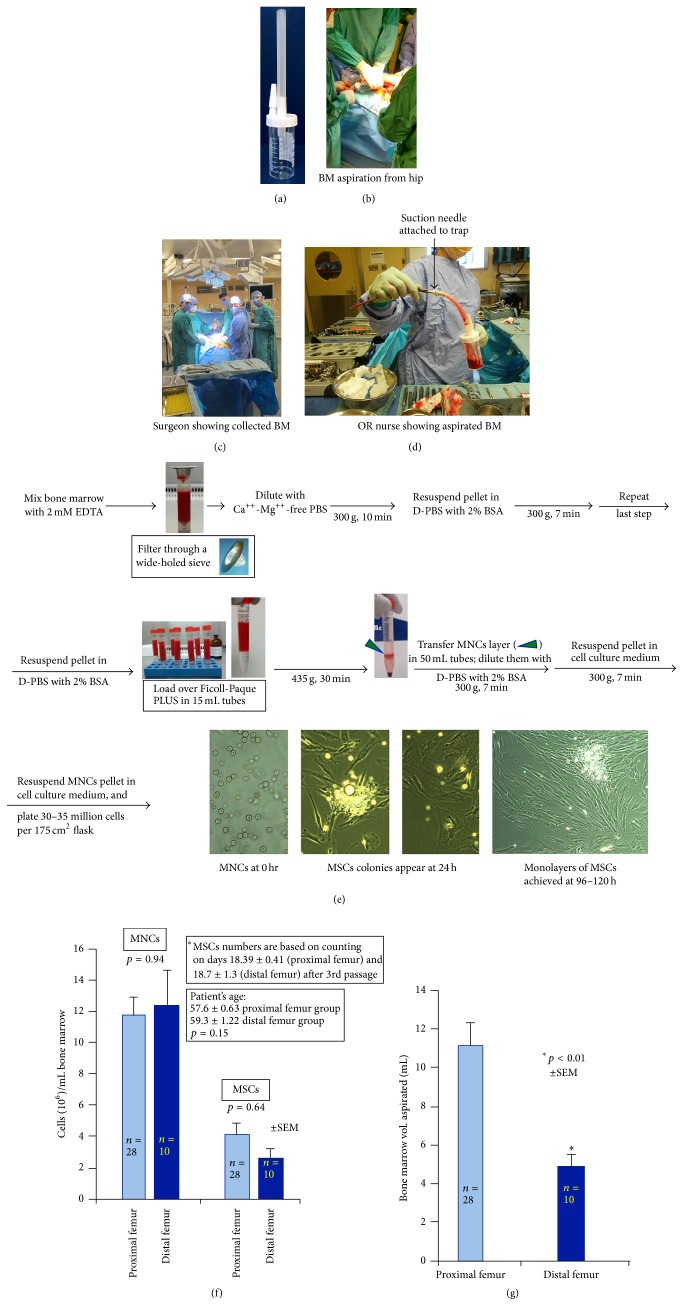
Bone marrow aspiration, mononuclear cells (MNCs) separation, and MSCs grown in cell culture (a–e). A specimen trap used for the collection of bone marrow (a). An orthopaedic surgeon aspirating bone marrow from the femur of a patient undergoing THA (b). The surgeon showing the specimen trap after aspiration of bone marrow (c). An OR nurse showing the full view of the specimen trap along with the suction needle and the collected bone marrow (d). A flow diagram showing the procedure of MNCs separation from the bone marrow and the establishment of MSCs in cell culture (e). A graph presenting recovery of mononuclear cells (MNCs) per mL bone marrow aspirated from proximal and distal femur from the patients undergoing THA and TKA, respectively (left); a graph presenting the number of viable MSCs (expanded up to passage 3) per mL bone marrow aspirated from proximal and distal femur from the patients undergoing THA and TKA, respectively (right) (f); a graph presenting the volume of bone marrow recovered from the proximal and distal femur from the patients undergoing THA and TKA for the purpose of MNCs separation (g).

Research Ethics Board at the University Health Network, Toronto, approved the research study protocol. Bone marrow was retrieved from patients (age group, 55–65 years) undergoing TKA or THA with their consent. Briefly, bone marrow was aspirated from the femur cavity by gliding a suction needle (13′′) attached at the rubber tubing of the specimen trap (BW406, Cardinal Health) containing 2 mL of heparin (2000 IU; DIN-453811, Heparin Leo®, Leo Pharma, Figure 1(a)). The nozzle of the trap was attached to vacuum system (Figures 1(b)–1(d)). The trap containing bone marrow was then transported to the laboratory at room temperature (RT) within 60 min.

### 2.2. Isolation of MSCs from Bone Marrow Aspirate

The separation of MSCs from bone marrow is shown in a flow diagram (Figure 1(e)). Bone marrow was diluted with 2 mM EDTA at 1 : 1 ratio (2 mM EDTA prepared from AM9260G, EDTA, 0.5 M, and pH 8.0, Life technologies in Ca^++^-Mg^++^-free PBS) followed by shaking the contents of the container 10–15 times. The diluted marrow was filtered through a wide-holed iron sieve (pore size: 1.25 × 1.25 mm^2^) to remove any fibrotic tissue and bone particles, further diluted at 1 : 3 with Ca^++^-Mg^++^-free phosphate-buffered saline (D8537, Sigma-Aldrich), and centrifuged at 300 g for 10 min in 50 mL tubes. The upper fat layer in the centrifuge tube was discarded. The large middle layer of supernatant was carefully aspirated and discarded. The bottom red colored pellet of each tube was resuspended in 35 mL D-PBS (D8662, Dulbecco's Phosphate-Buffered Saline, Sigma-Aldrich) containing 2% bovine serum albumin (A9418, Sigma-Aldrich) and subsequently centrifuged at 300 g for 7 min. This step was repeated once to wash off residual EDTA. Finally, the pellet was resuspended in D-PBS with 2% BSA in a volume equal to original volume of bone marrow. A total of 5–7 mL of pellet suspension was layered on the top of 4 mL Ficoll-Paque PLUS (17-1440-02, GE Healthcare) in 15 mL tubes and centrifuged at 435 g for 30 min to achieve gradients. The middle gradient layer rich in mononuclear cells (MNCs) was carefully removed, diluted in D-PBS with 2% BSA, and centrifuged at 300 g for 7 min. The washing step was repeated with MSCs culture medium. The MNCs pellet was resuspended in MSCs culture medium. Cells were counted and checked for viability and viable cells are presented in Figure 1(f). A total of 30–35 million MNCs were plated in 175 cm^2^ tissue culture flask (353112, BD Falcon) containing 40 mL MSCs culture medium in a humidified incubator with 5% CO_2_. MSCs colonies were detected at 24 h and became larger within 48–72 h. Media were changed every third day. Once 70–80% were confluent, the dishes were trypsinized with TrypLE*™* Select CTS*™* (A12859, Gibco® by Life Technologies) and placed in culture for next passage. The MSCs medium consisted of DMEM (11054, Gibco by Life Technologies), 1% GlutaMAX*™* CTS*™* (100x, A12860, Gibco by Life Technologies), and 1% penicillin-streptomycin (100x, P4333, Sigma-Aldrich) supplemented with 10% heat-inactivated FBS (SH30070.03, HyClone Fetal Bovine Serum, Thomas Scientific). MSCs' characteristic positive and negative markers and their ability to differentiate into three different lineages were evaluated.

The number of bone marrow samples aspirated from patients undergoing THA and TKA was 28 and 10, respectively. The male and female ratio was 1 : 1 in each group. An average number of viable MNCs per mL bone marrow were 11.74 ± 1.21 × 10^6^ (range: 1.7 × 10^6^ to 29.77 × 10^6^) and 12.37 ± 2.28 × 10^6^ (range: 2.36 × 10^6^ to 30.33 × 10^6^) from proximal and distal femur from patients undergoing THA and TKA, respectively (Figure 1(f)). There was no significant difference in the recovery of MNCs from bone marrow aspirated from these two sites (*p* = 0.94). Average age of the patient undergoing THA and TKA was 57.60 ± 0.63 (range: 51–63) and 59.3 ± 1.22 (range: 51–64) years (*p* = 0.15). Very interestingly, the volume of bone marrow aspirated from proximal femur was 2.29-fold higher than from distal femur (11.17 ± 1.15 versus 4.87 ± 0.63 mL per aspiration, *p* < 0.01; Figure 1(g)). The expanded numbers of viable MSCs per mL bone marrow at passage 3 (on day 18.39 ± 0.41 versus 18.7 ± 1.13, *p* = 1, in proximal and distal femur, resp.), from proximal and distal femur [4.1 ± 0.72 × 10^6^ (range: 0 to 14.22 × 10^6^) versus 2.56 ± 0.69 × 10^6^ (range: 0 to 8.33 × 10^6^)], did not differ from each other (*p* = 0.64; Figure 1(f)).

### 2.3. Characterization of MSCs by Immunocytochemistry (ICC)

MSCs were characterized for their specific positive and negative cell surface markers by immunofluorescence technique using a kit (SCR067, Human MSC Kit, Millipore). Manufacturer's instructions were followed. Positive cell markers included antibodies directed against cell surface antigens present on mesenchymal stem cells such as CD44, CD90, CD146, and STRO-1. Negative markers included those specific to cells of the hematopoietic lineage CD14 (present on leukocytes) and CD19 (present on B-lymphocytes). In brief, MSCs at passage 3 (1–1.2 × 10^4^ cells/0.5 mL in culture medium in 0.69 cm^2^ well) were plated in the wells of 8-chamber tissue culture glass slides (CA62405-178, BD Falcon). At 48 h, media were carefully aspirated. Adherent cells were fixed in freshly diluted 4% PFA (15710, 16% paraformaldehyde, EM Sciences) in D-PBS for 40 min at RT. Fixed cells were rinsed in D-PBS (3x, 5 min), treated with a nonpermeable blocking buffer (5% donkey serum in D-PBS for 2 h), replaced by primary antibody (diluted in blocking buffer), and incubated overnight (O/N) at 4°C in a humidified box. The following antibodies were used: mouse anti-H-CAM; mouse anti-THY-1 (CD90); mouse anti-MCAM (CD146); mouse anti-STRO-1; mouse anti-CD19; mouse anti-CD14. In negative control wells, equivalent concentrations of only mouse IgG or IgM were added. The cells were washed with D-PBS (2x, 5 min) and blocking buffer (2x, 5 min) and left in blocking buffer for 30 min. Secondary antibodies, donkey anti-mouse IgG Cy3 conjugated (AP192C; 1 : 250, Millipore) or donkey anti-mouse IgG Alexa Fluor® 488 conjugated (715-545-151, Jackson Immunoresearch; 1 : 500), diluted in blocking buffer, were added and incubated for 2 h at RT. Cells were washed with D-PBS (4x, 5 min), counterstained with DAPI in D-PBS solution, and washed, and slides were mounted in antifading mounting medium. Slides were visualized under Olympus FluoView 1000 laser scanning confocal microscope. It is generally accepted that cells that express CD44, CD90, CD146, and STRO-1 and but do not express CD14 and CD19 represent a MSC population [4–9]. ICC assays were repeated with MSCs samples from 4 donors and representative results are presented.

### 2.4. Flow Cytometry

MSCs were also characterized by flow cytometry after three cell culture passages. MSCs were stained with the following commercially available antibodies: CD105-PE (PE mouse anti-human CD105, Cat. 323205, BioLegends); CD34-PE-Cy7 (mouse PE-Cyanine7 anti-human CD34, Cat. 25-0349-42, eBioscience); CD45-APC (mouse APC anti-human CD45, Cat. 555485, BD Pharmingen*™*); CD19-PE-Cy  7 (mouse PE-Cy*™*7 anti-human CD19, Cat. 557835, BD Pharmingen); CD73-APC (mouse APC anti-human CD73, Cat. 560847, BD Pharmingen); CD146-PE (mouse PE anti-human CD146, Cat. 550315, BD Pharmingen); CD14-PE (mouse PE anti-human C14 antibody, Cat. 301805, BioLegend); CD90-APC (mouse APC anti-human CD90, Clone 5E10 (RUO), Cat. 559869, BD Biosciences). Antibody staining was performed at 4°C in PBS containing 5% (v/v) fetal calf serum (FCS). Approximately 50,000–100,000 cells were stained per well. Cells were acquired using a LSR II flow cytometer (Becton Dickinson). Analysis was performed using FlowJo (Tree Star).

### 2.5. Characterization of MSCs by Differentiation Assays

Differentiation assays were repeated with MSCs samples from 4 donors and representative results are presented. Chondrogenesis was studied in more detail.

#### 2.5.1. Adipogenesis

Adipogenesis of MSCs was conducted using a kit (A10070-01, STEMPRO®, Thermo Fisher Scientific) following the manufacturer's instructions. In brief, MSCs (38,000) were seeded per well into a 12-well cell culture plate (3513, Costar®, CORNING) at a density of 1 × 10^4^ cells/cm^2^ in MSCs medium at 37°C in a humidified atmosphere at 5% CO_2_. After 3 h, media were replaced with prewarmed adipogenesis differentiation medium and continued incubation (day 0). MSCs continued to undergo limited expansion as they differentiated under adipogenic conditions. Adipogenic medium was replaced every 3 days. On day 9, the cells were stained with Oil Red O and photographed.

*Oil Red O Staining*. Oil Red O (Solvent Red 27, Sudan Red 5B) is a lysochrome (fat-soluble dye) diazo dye used for staining of neutral triglycerides and lipids on frozen sections and some lipoproteins on paraffin sections. It has the appearance of a red powder. To evaluate the adipogenic differentiation, Oil Red O (O0625, Sigma-Aldrich) staining of the cytoplasmic droplets of neutral lipids in the differentiated cells was performed. The cells were rinsed with D-PBS and fixed with 10% NBF (neutral buffered formalin, HT501128, Sigma Aldrich) for 40 min. The working solution of Oil Red O was prepared by mixing three parts of Oil Red O stock solution (0.3 g/mL Oil Red O powder in 99% isopropanol) with two parts of dH_2_O, incubated for 10 min and filtered through Whatman paper. After rinsing with distilled water (dH_2_O) once and treating with 60% isopropanol for 5 min, the cells were treated with 2 mL Oil Red O working solution for 5 min at RT. Stained cells were washed with tap water until water rinses off clear, stained with 2 mL of Meyer's hematoxylin (2 min), and washed with tap water until water rinses off clear and photographed while being still under water using Zeiss Discovery V8 Stereomicroscope.

#### 2.5.2. Osteogenesis

Osteogenesis of MSCs was conducted using a kit (A10072-01, STEMPRO, Thermo Fisher Scientific) following the manufacturer's instructions. In brief, MSCs (19,000) were seeded per well into a 12-well cell culture plate at a density of 0.5 × 10^4^ cells/cm^2^ in MSCs medium at 37°C in a humidified atmosphere at 5% CO_2_. After 3 h, media were replaced with prewarmed complete osteogenesis differentiation medium and continued incubation (day 0). MSCs continued to undergo limited expansion as they differentiated under osteogenic conditions. Media were replaced every 3 days. On day 20, the cells were stained with Alizarin Red S and photographed.

*Alizarin Red S Staining*. For osteogenesis, the deposition of calcium phosphate is an indication of MSCs differentiation into osteoblast and hence* in vitro* bone formation. Medium in the cell culture wells was removed; cells were washed with Ca^++^-Mg^++^-free D-PBS and then fixed in 2 mL 10% NBF (40 min). Cells were washed with dH_2_O, stained with Alizarin Red S (2%, pH 4.3; A5533, Sigma-Aldrich; 2-3 min), and washed with dH2O (3x). Stained cells were photographed while being still under water using Zeiss Discovery V8 Stereomicroscope.

#### 2.5.3. Chondrogenesis

For chondrogenesis assays, micromass cultures were established. Cells were seeded in a total volume of 30 *μ*L onto a dry flat-bottomed 24-well plate (3526, Costar 24-Well Clear TC-Treated Multiple Well Plates) at a density of ~25 × 10^6^ MSCs/mL. The plate was placed in the humidified CO_2_ incubator at 37°C for 2 h and then photographed showing the high density of MSCs at the beginning of chondrogenesis. Additional chondrogenesis media (0.75 mL) were added to each well. Media were changed every 48 h. Micromass (mm) cartilages were retrieved at weeks 1, 2, 3, and 4. At each time point, they were photographed and fixed in 4% PFA for 24 h at 4°C. The micromass cartilages were washed in D-PBS, transferred in 70% ethanol, and processed for histology. The paraffin sections (4 *μ*m) were assessed for collagen by Masson's trichrome staining, proteoglycans by safranin O staining, localization of COL II, COL I, COL X, aggrecan, and COL VI by immunohistochemistry, and apoptosis by AopTag® Peroxidase* In Situ* Apoptosis Detection Kit. Micromass cartilages were separately processed for transmission electron microscopy for ultrastructure of chondrocytes and extracellular matrix.

*(1) Chondrogenesis Medium*. Chondrogenesis medium was prepared by mixing incomplete chondrogenesis medium with TGF*β*3 at a concentration of 10 ng/mL. TGF*β*3 powder (243-B3, recombinant human TGF-beta 3, R&D Systems) was reconstituted as stock solution as per manufacturer's instructions. The stock solution (20 ng/*μ*L) was stored in 25 *μ*L aliquots at −70°C. Complete chondrogenesis medium was prepared by adding 500 ng TGF*β*3 (25 *μ*L stock solution) to 50 mL incomplete chondrogenesis medium. The complete chondrogenesis medium was used within 2 days. The incomplete chondrogenesis medium was prepared as shown in Table 1 and was stored up to one week at 4°C.

*(2) Masson's Trichrome Staining*. Slides with paraffin tissue sections were heated at 58–60°C for 60 min. Tissue paraffin sections were deparaffinized and hydrated as described earlier in the text. The slides were placed in prewarmed 0.2% chromic acid at 60°C (5 min), rinsed in tap H_2_O (5x), stained in Weigert's iron hematoxylin (10 min), washed in tap H_2_O till blue coloration leaches off, differentiated in 1% acetic acid (2x, 1 sec), washed in running tap H_2_O (2 min), treated with 1% Biebrich Scarlet (AC402221000, Fisher Scientific, in acetic acid) for 1 min, rinsed in running tap water (2 min), treated with 5% phosphotungstic acid/phosphomolybdic acid (A237-100, A248-500, Fisher Scientific) for 1 min, treated in 1% light green SF (P399-03, JT Baker), and differentiated in 1% acetic acid and rinsed in tap water (5x). The sections were dehydrated in increasing concentration of EtOH (70, 95, and 100%, 2x, 3 min) and cleared with Histo-Clear II® (3x, 3 min). The slides were semi-air-dried and mounted using Omnimount*™* adhesive medium. As the name implies, this staining employs selectively staining muscle, collagen fibers, fibrin, and erythrocytes. Nuclei stain black, cytoplasm, muscle, and erythrocytes stain red, and collagen stains blue.

*(3) Safranin O Staining*. Safranin O staining in micromass cartilages localizes proteoglycans. Slides with paraffin tissue sections were heated at 58–60°C for 60 min. Paraffin sections at RT were deparaffinized in Histo-Clear II (3x, 3 min, 64111-01, EM Sciences) and hydrated in decreasing concentration of ethanol [EtOH, 100%, 95%, 70%, and 0% (dH_2_O), 2x, 3 min each] and finally placed in dH_2_O. The sections were stained in Weigert's iron hematoxylin (5 min) and rinsed in changes of dH_2_O till leaching of blue coloration stopped. Sections were differentiated in 1% acid-alcohol (2 secs), rinsed in dH_2_O (4x), and treated with 0.02% Fast green (15 min), 1% acetic acid (10 secs), and 1% safranin O (20800, EM Sciences) for 10 min. The sections were dehydrated in 95% EtOH (4x, 15 secs) and 100% EtOH (4x, 15 secs) and cleared in Histo-Clear II (3x, 3 min) and mounted in Omnimount mounting medium (17997, EM sciences). Proteoglycans stain red, cytoplasm stains gray green, and nuclei stain black. Knee paraffin section (4 *μ*m thick) from a rat was stained for safranin O staining as a positive control.

*(4) Immunohistochemistry (IHC)*. Micromass cartilages were characterized by localizing specific antigens by IHC. Slides were heated at 58–60°C for 60 min. The slides at RT were deparaffinized and hydrated as described earlier in the text. The sections went through antigen retrieval process. Sections were incubated with blocking buffer for 30 min and then with primary antibody (Table 2) overnight at 4°C, followed by step A or B as shown below depending upon the primary antibody used: (A) for COL II, COL X, COL I, and aggrecan, the sections were washed with PBS-T (4x, 5 min), treated with blocking buffer for 30 min, and incubated with secondary antibody (Table 2) for 30 min. The sections were washed with PBS-T (4x, 5 min) and incubated with ABC reagent [ABC reagent was prepared 30 min before incubation (PK-6100, Elite ABC-HRP kit, Vector)] for 30 min. (B) For COL VI, the sections were washed with PBS-T (4x, 5 min) and treated with Streptavidin-HRP (diluted in PBS-T, ab7403, abcam) for 30 min. Following step A or B, sections were washed with PBS-T (2x, 3 min; 4x, dH_2_O, 5 min) and brown color was developed using DAB substrate as described earlier. The sections were stained with Meyer's hematoxylin, washed, dehydrated, cleared, mounted, scanned, and photographed as described earlier. Paraffin sections from an adult human tracheal cartilage (CAR01, Pantomics, Inc., Richmond, CA), knee sections from an adult rat, and undecalcified limb and spine section from P1 mouse were used as positive controls where applicable.

*(5) Apoptosis*. Slides with paraffin tissue sections were heated at 58–60°C for 60 min. Apoptosis in generated micromass cartilage sections was determined by using a kit (7100, AopTag Peroxidase* In Situ* Apoptosis Detection Kit, Millipore) following manufacturer's instructions. During apoptosis, DNA fragmentation takes place and apoptotic bodies are rich in free OH^−^ ends. The kit is designed to label free OH^−^ ends by adding a mixture of digoxigenin-labeled and nonlabeled nucleotides in the presence of terminal deoxynucleotidyl transferase. The resulting oligomer added to apoptotic DNA fragment's ends allows the binding of anti-digoxigenin antibody conjugated to a peroxidase reporter molecule which was detected using 0.4 mL freshly diluted peroxidase substrate diaminobenzidine (DAB; Sk-4105, ImmPACT*™* DAB Substrate, Vector) that gives dark brown staining and the reaction was stopped by placing the slides in dH_2_O in a Coplin jar. The sections were stained with Meyer's hematoxylin, washed, dehydrated, cleared, and mounted as described earlier. The mounted slides were scanned at 20x using Aperio ScanScope XT. The images were opened in a PC computer using the image analysis software Aperio ImageScope (version 10). The images were saved as tif files. The mammary gland, undergoing involution at day 4 postpartum (S7115, Millipore, Canada), was used as positive control.

*(6) Transmission Electron Microscopy (TEM)*. Four-week micromass cartilages were evaluated by TEM at their upper middle zone. The* in vitro* generated cartilages were fixed in universal buffer for 48 h at 4°C. The tissues were cut into smaller pieces, washed in Millonig's buffer (3x), postfixed in 1% osmium tetroxide in 0.1 M sodium cacodylate (pH 7.4), and washed again with Millonig's buffer (3x). Tissues were dehydrated in increasing concentrations of ethanol up to 100%, transferred to 100% acetone, infiltrated in acetone/epon (1 : 1), transferred in 100% epon, and embedded in beam molds and polymerized for 2 days at 70°C. Semithin sections at 1 *μ*m were cut for toluidine blue staining. For TEM, 90°A sections were cut. The grids were stained with uranyl acetate/lead citrate. The sections were examined using a JOEL JEM-1011 electron microscope.

## 3. Results

### 3.1. Bone Marrow Aspiration and MSCs Characterization

We presented a simplified procedure for the aspiration of bone marrow from the femur of patients undergoing TKA and THA (Figures 1(a)–1(d)). The bone marrow was collected without clotting or with minimal clotting in all the aspirations we conducted so far using this procedure. Further, we showed that fatty material was easy to remove in the first step of centrifugation before loading the marrow over Ficoll-Paque PLUS for gradient formation (Figure 1(e)). Passing the bone marrow through wide-holed sieve proved to be a useful step since it removed any bone spur or fibrous tissue or any clotted pieces of bone marrow that are likely to disrupt gradient separation of mononuclear cells (MNCs). As far as we know, this is the first of its kind in a simple protocol form that we are reporting here. The volume of bone marrow retrieved from femur varied from patient to patient (≈2–20 mL). Here, we report MSCs isolation from bone marrow aspirated from proximal femur from 28 THA patients (M : F; 1 : 1) and distal femur from 10 TKA patients (M : F; 1 : 1).

**Figure 2:**
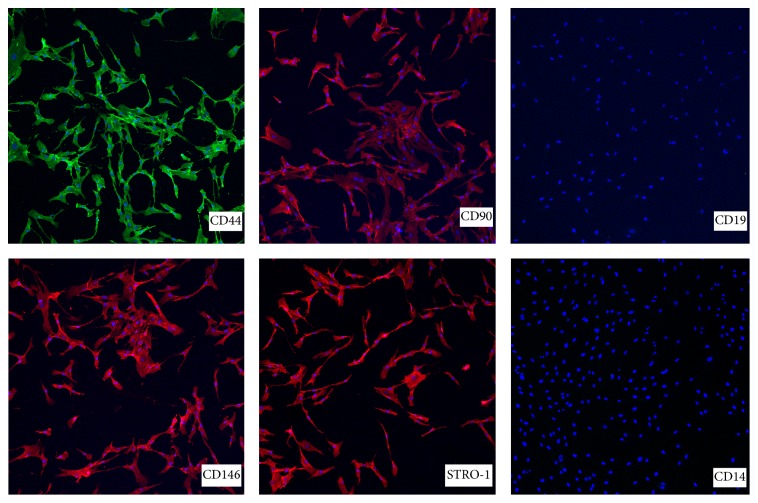
Immunocytochemistry of cell surface markers for human bone marrow-derived MSCs. Positive markers (CD44, CD90, CD146, and STRO-1) and negative markers (CD19 and CD14) are shown as labeled in each panel. CD44 is stained green with Alexa Fluor 488 conjugated secondary antibody, whereas the secondary antibody for the rest of the panels was conjugated with Cy3 and that showed red color in positive markers or absence of red colored labeling in negative markers. Nuclei stained blue with DAPI in all the panels.

**Figure 3:**
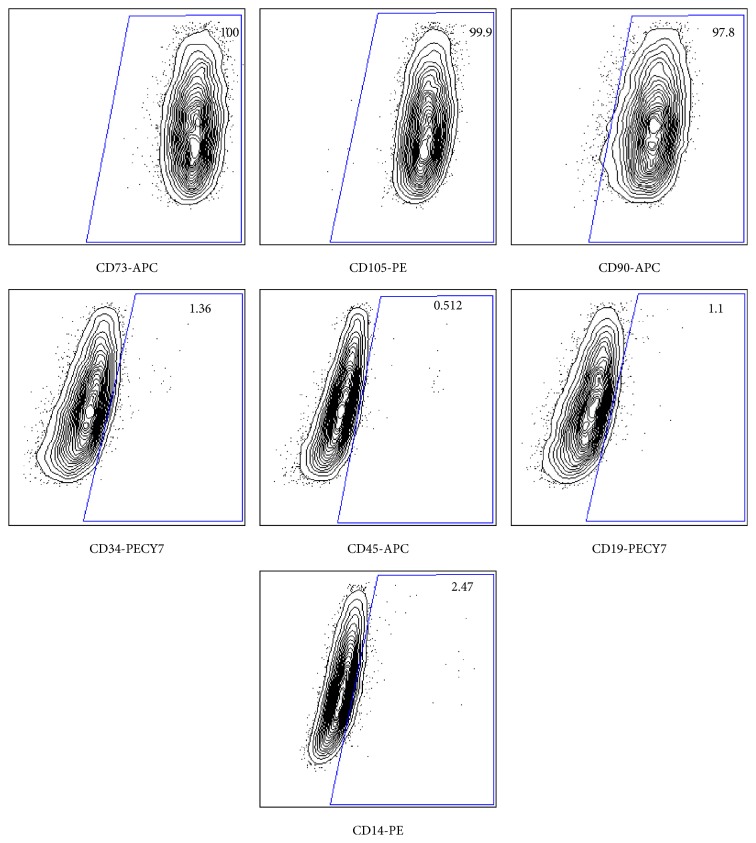
Flow cytometry of human bone marrow-derived MSCs indicating the percentage of each marker in each panel and name of conjugated antibody at the bottom of each panel. C73, CD105, and CD90 shown as positive markers and CD34, CD45, CD19, and CD14 shown as negative markers.

**Figure 4:**
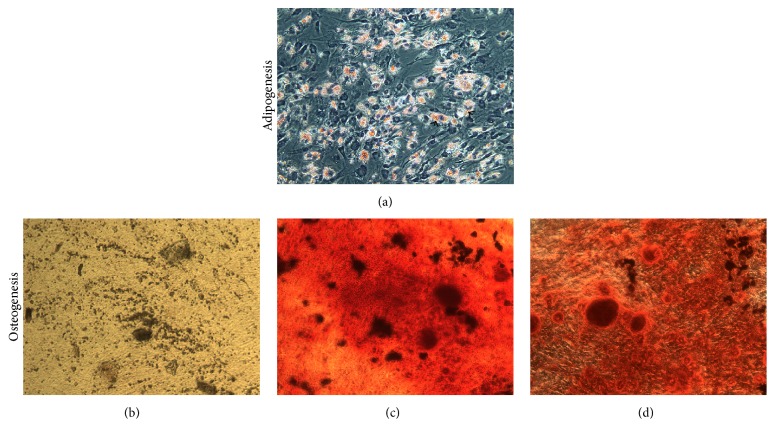
Bone marrow-derived MSCs differentiating into adipocytes (a) and osteoblasts (b–d). Lipid droplets are visible in adipocytes (arrow, a) and mineralization: partly dark (unstained, b), and Alizarin Red-stained bright field (c) and phase-contrast (d) calcium deposits developed in osteoblasts.

**Figure 5:**
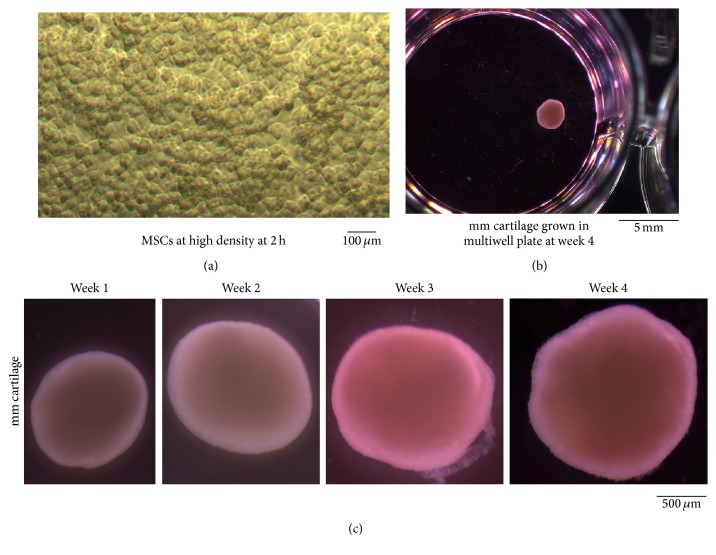
Chondrogenic differentiation of bone marrow-derived MSCs, and generation of micromass cartilages (a–c). A high density of MSCs at 2 h shows MSCs condensation at the beginning of chondrogenesis (a). A micromass cartilage, shown in a 24-well plate, at week 4 of chondrogenesis (b). Micromass cartilage, at weeks 1, 2, 3, and 4, shows a gradual increase in size with time (c).

The MSCs that were obtained using this procedure showed positive markers (CD44, CD73, CD105, CD90, CD146, and STRO-1) and negative markers (CD14, CD19, CD34, and CD45) as shown by immunofluorescence and/or flow cytometry (Figures 2 and 3). MSCs were able to differentiate into three lineages* in vitro*: adipocytes (Figure 4(a)), osteoblasts (Figures 4(b)–4(d)), and chondrocytes (Figure 5). High density MSCs that were plated in 30 *μ*L drops (Figure 5(a)) underwent chondrogenic differentiation and the resulting micromass cartilage in 24-well plate (Figure 5(b)) increased in size from week 1 through week 4 (≈2 mm in diameter at week 4; Figure 5(c)).

### 3.2. Micromass Chondrogenesis and Assessment

**Figure 6:**
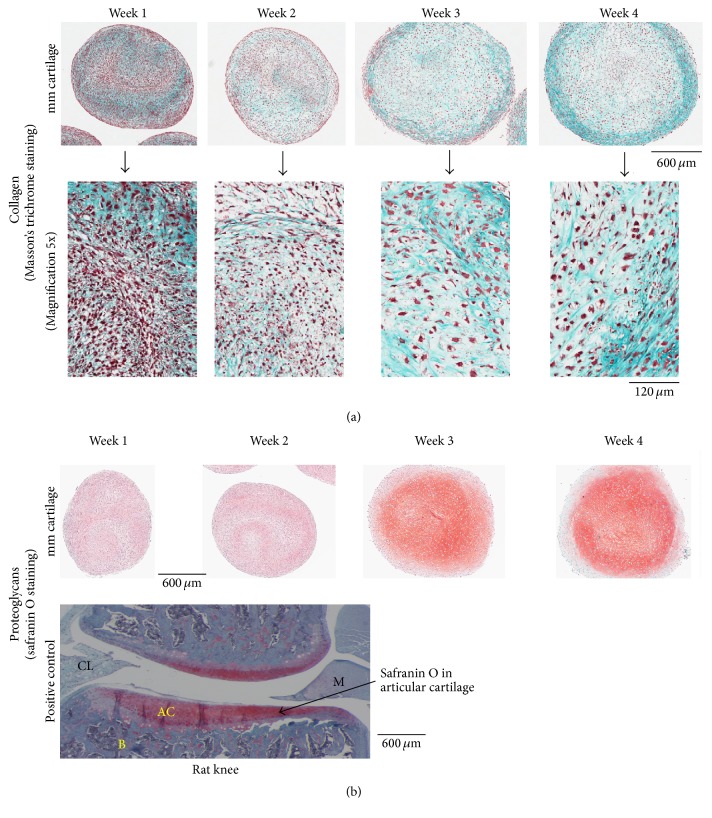
Collagen (blue colored fibers) in micromass cartilages as shown by Masson's trichrome staining (a), and proteoglycans (orange-red color) in micromass cartilages as shown by safranin O staining (b). Higher magnification shows more details that collagen (blue colored fibers), cell size, and intercellular distance increase from week 1 to 4 in micromass cartilages (a). Proteoglycans were dominant at weeks 3 and 4 and were weakly expressed at weeks 1 and 2 (b). Rat knee articular cartilage served as a positive control for safranin O staining (b). M, meniscus; AC, articular cartilage; B, bone; mm, micromass; CL, Cruciate Ligament.

**Figure 7:**
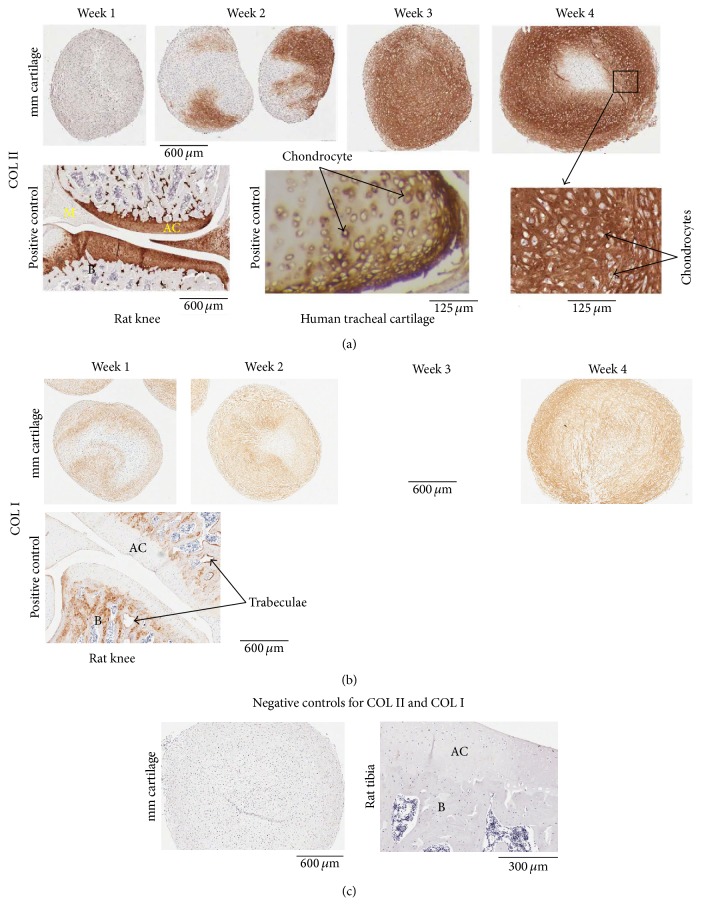
IHC of COL II (a) and COL I (b) in micromass cartilages. COL II expressed at weeks 2–4 (a), and COL I expressed at weeks 1–4 (week 3 not shown, b), in micromass cartilages. Articular cartilage of rat knee and human tracheal cartilage served as positive control for COL II; higher magnification of week 4 micromass cartilage shows that it is present in ECM (a). COL I is absent in native articular cartilage in positive control and is present in bone only, whereas COL I is expressed at low level in the bioengineered micromass cartilage (b). Micromass cartilage and rat tibia showed no staining when primary antibody was missing and only mouse IgG was present, which served as negative control (c). M, meniscus; AC, articular cartilage; B, bone; mm, micromass.

**Figure 8:**
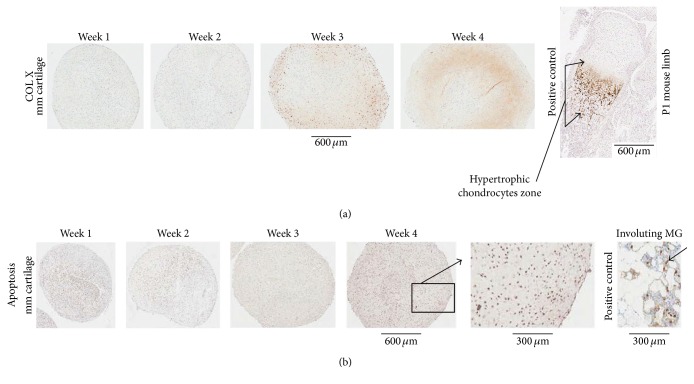
IHC of COL X shows the presence of weak signal in week 3 and week 4 micromass cartilage (a), whereas apoptosis assays show no apoptosis in micromass cartilage (b). Mouse decalcified P1 limb showed hypertrophic chondrocytes zone (arrows) as positive control for COL X (a). Mouse involuting MG (mammary gland) at postpartum day 4 showed apoptotic cells in mammary epithelium (arrow) that served as positive control, whereas no apoptotic cell was found at week 4 in micromass cartilage compared with positive control. mm, micromass.

Masson's trichrome staining showed that collagen (blue colored fibrils), intercellular distance, and chondrocyte size increased from week 1 to week 4 in micromass cartilages (Figure 6(a)). Safranin O staining showed the presence of proteoglycans at weeks 1 and 2 that became abundant at weeks 3 and 4 (Figure 6(b)). Rat knee served as a positive control for safranin O staining that showed proteoglycans in articular cartilage (Figure 6(b)). COL II began to be expressed at week 2 and became prominent at subsequent time points (weeks 3 and 4) in micromass cartilages (Figure 7(a)). Rat knee articular cartilage along with meniscal cartilage and human tracheal cartilage served as positive staining for COL II (Figure 7(a)). Col II was present around chondrocytes and in intercellular matrix (Figure 7(a)). COL I was expressed at all of the time points in micromass cartilage (week 3 not shown, Figure 7(b)). Rat knee served as positive control for COL I, the expression of which was shown in trabeculae only, and the native articular cartilage did not express COL I (Figure 7(b)). Negative controls are shown in Figure 7(c). COL X showed weak expression at weeks 3 and 4 and that is a sign of low level of hypertrophy of chondrocytes (Figure 8(a)). Hypertrophic chondrocyte zone in undecalcified limb section of P1 mouse was used as positive control (Figure 8(a)). Micromass cartilages did not show apoptosis (Figure 8(b)). Mouse involuting mammary gland (day 4 postpartum) served as positive control for apoptosis (Figure 8(b)).

**Figure 9:**
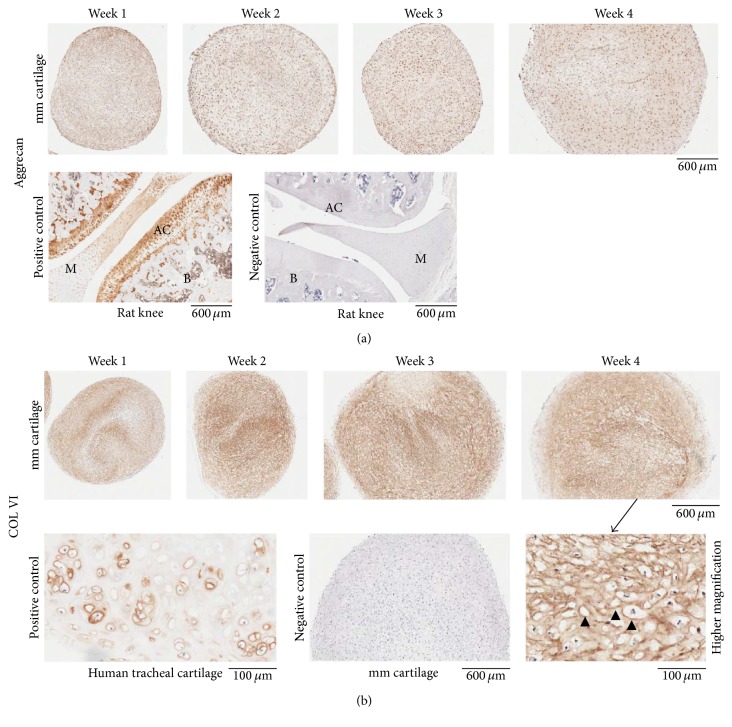
IHC showed that micromass cartilage expressed aggrecan and COL VI at all the time points (weeks 1–4, a, b). Articular cartilage of rat knee expressed aggrecan and served as positive control (a). Human tracheal cartilage served as positive control for COL VI and showed that COL VI is pericellular in location in native cartilage (b). Higher magnification of week 4 bioengineered micromass cartilage also shows the pericellular localization of COL VI (arrowheads, b). Rat knee (a) and micromass cartilage (b) were used as negative control with rabbit IgG polyclonal and without primary antibody. M, meniscus; AC, articular cartilage; B, bone; mm, micromass.

**Figure 10:**
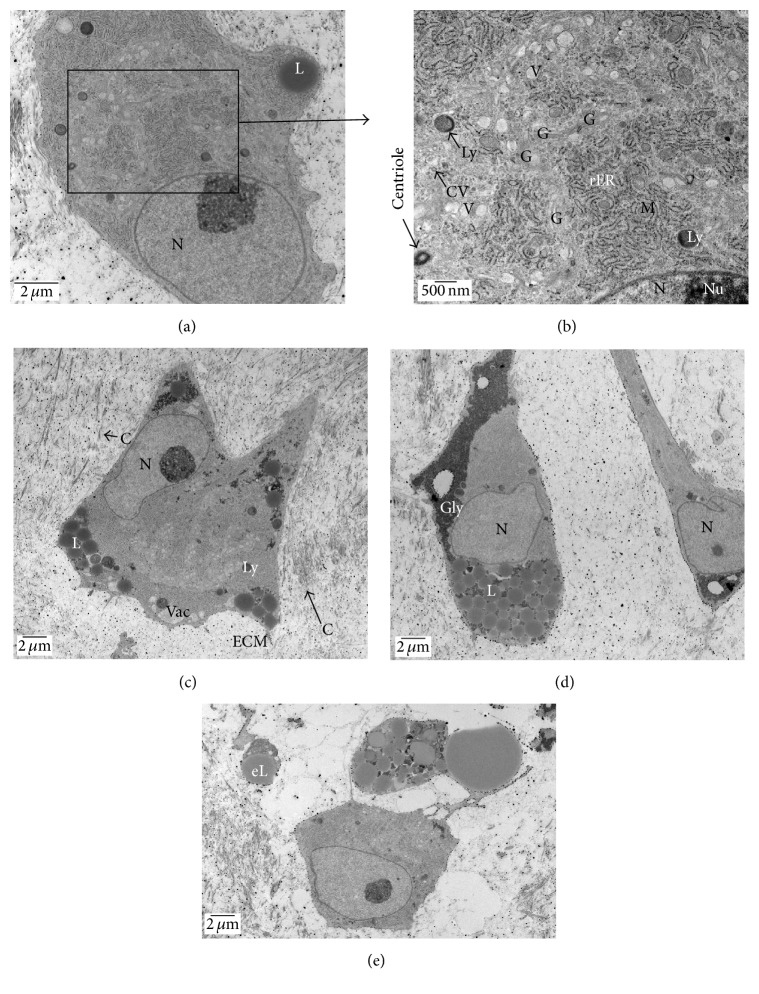
TEM from upper middle area of micromass cartilage at week 4 represents normal structure of chondrocytes in bioengineered cartilage (a–e). Panel (b) exhibits the higher magnification of panel (a). Chondrocytes appear to be metabolically active with extensive network of Golgi, well-developed rER, with euchromatic nucleus, usually with the presence of nucleolus, a number of secretory vesicles and coated vesicles, a number of lipid droplets, the presence of glycogen, a number of mitochondria, and the presence of lysosomes. Centriole is visible occasionally. Intercellular regions show the presence of collagen fibers and extracellular matrix. Nucleus is oval to irregular in shape (a, c–e). L: lipid droplet, eL: extracellular lipid droplet, M: mitochondrion, rER: rough endoplasmic reticulum, Ly: lysosome, G: Golgi network, V: vesicle, CV: coated vesicle, N: nucleus, Nu: nucleolus, Vac: vacuole, C: collagen fibers, and ECM: extracellular matrix.

Aggrecan and COL VI showed expression in micromass cartilages (Figures 9(a) and 9(b)). Rat knee articular cartilage served as positive control for aggrecan (Figure 9(a)). Human tracheal cartilage served as positive control for COL VI; higher magnification shows that the COL VI is centered at pericellular regions of chondrocytes or lacunae containing chondrocytes (Figure 9(b)). Transmission electron microscopy images of micromass cartilage from the upper middle area at week 4 are shown (Figures 10(a)–10(e)) and that indicates that chondrocytes are normal in week 4's micromass cartilage. Chondrocytes appears to be metabolically active with extensive Golgi network, well-developed rough endoplasmic reticulum (rER), euchromatin nucleus with nucleolus, a large number of secretory vesicles and coated vesicles, a number of lipid droplets, and glycogen, mitochondria, and lysosomes. Centriole is present. Extracellular matrix (ECM) shows collagen fibers (Figures 10(a)–10(e)).

### 3.3. Statistical Analysis

The aspirable volume of bone marrow from femur, number of viable MNCs collected from bone marrow, patient age, number of viable MSCs at passage 3 after expansion, and the day at which successful expansion of viable MSCs was achieved were compared between proximal (*n* = 28) and distal (*n* = 10) femur bone marrow aspirations by using the Wilcoxon test for paired samples. A value of *p* < 0.05 was considered as statistically significant, with a 95% confidence interval.

## 4. Discussion

### 4.1. Bone Marrow Aspiration and MSCs Characterization

Bone marrow is a flexible tissue in the interior of bones. On an average, bone marrow constitutes 4% of the total body mass of humans. Bone marrow constitutes hematopoietic and lymphatic system. Bone marrow transplants can be conducted to treat severe diseases of the bone marrow, including certain forms of cancer such as leukemia [10]. Additionally, bone marrow stem cells can be differentiated into other lineages and used to treat diseases [11]. The posterior iliac crest is a readily accessible site for bone marrow aspiration that is safe [1]. In general, for biopsy and clinical diagnosis, clinicians collect bone marrow from posterior and anterior iliac crest and lumbar pedicle. Alternatively, bone marrow can be aspirated from the femur of patients undergoing TKA or THA. There are reports of researchers using bone marrow aspirated from patients though standard operating room protocol has never been published [2, 3]. Bone marrow aspiration procedure from patients may vary from surgeon to surgeon and may lead to partial or full clotting of bone marrow within seconds. By introducing this procedure, the bone marrow is aspirated directly into the specimen trap and that gets mixed with anticoagulant instantly resulting in minimum or no clotting of bone marrow (Figures 1(b)–1(d)). The bone marrow provides acceptable number of MNCs. Second, we introduced a step of filtering diluted bone marrow through wide-holed sieve to remove any fibrous tissue or small piece of bone in the marrow (Figure 1(e)). The bone marrow aspiration from patients undergoing TKA and THA is a very economical procedure. Purchasing bone marrow-derived MSCs from a standard biotechnology company can be very expensive and can cost up to 2,000 USD per million MSCs at passage 2. MSCs isolated from patients' bone marrow were able to undergo trilineage differentiation (adipogenesis, osteogenesis, and chondrogenesis) and exhibited specific positive and negative cell surface markers (Figures 2–5) similar to that reported earlier [12, 13]. The chondrogenesis was further assessed in detail (Figures 6–10).

An interesting study was published recently by Narbona-Carceles and colleagues [14], in which the authors aspirated bone marrow from three different sites from the patients prior to TKA in the same operating room for the purpose of isolation and characterization of MSCs. They obtained bone marrow aspirates through bone puncture of the iliac crest, distal femur, and proximal tibia, and the samples were aspirated by a single investigator. To avoid hemodilution of the aspirate, the depth and angle of the trocar were changed after every 2 mL of material had been aspirated. Samples were dispensed in heparinized tubes. We aspirated bone marrow from a single site either proximal or distal femur from patients undergoing THA and TKA, respectively. Hence, we will exclude iliac crest for comparison purpose. We did not use a separate session for bone marrow aspiration from patients in the operating room before THA or TKA surgery; rather, we aspirated bone marrow during surgical procedure. Since our procedure of aspiration was simple, any surgeon on operating duty was suitable for these aspirations. The bone marrow was aspirated from the femur cavity by gliding a suction needle (13′′) attached at the rubber tubing of the specimen trap containing heparin. The nozzle of the trap was attached to vacuum system as shown in Figure 1.

The procedure we devised, for the bone marrow aspiration from femur during THA or TKA, is simple, ethical, and novel due to the following reasons and has advantages over bone puncturing procedure in the knee [14]. (1) In our procedure, there was no need of special-skilled surgeon because of its simplicity. Any available surgeon was able to do aspiration. (2) In the event, bone marrow aspiration is proposed to be collected by bone puncturing method of knee [14] or hip in a separate session in the patients undergoing TKA or THA; an ethical concern would be evident as compared to aspiration from open wound during THA or TKA. Also, patients may hesitate to provide consent for puncturing of knee or hip just before their painful THA or TKA surgery due to the assumptive pain perception [15]. (3) An aspiration of bone marrow, from multiple points of a bone marrow site, is considered as better aspiration as compared to that from a single point [16]. The bone puncturing method and maneuvering of needle at different depths [14] in femur may require multiple punctures of bone to fulfill that requirement. That may add additional time on patient under anesthesia as compared to the current procedure. The aspiration of bone marrow from an open wound procedure during THA or TKA takes ≈10–15 secs. (4) In the bone puncturing method [14], the bone marrow is collected in a syringe and then dispensed into a container with an anticoagulant. This procedure may be inefficient for stopping part of bone marrow clotting. In addition, if the multiple puncturing is required, that will be of additional concern, in this regard. This problem does not exist in our current procedure since bone marrow is aspirated and mixed instantly with heparin, already present in the specimen trap, under the influence of suction pressure. (5) In bone puncturing method of bone marrow collection from femur, there are more chances of getting bone marrow contaminated with blood or other tissue fluids or tissues pieces depending upon the accuracy of needle insertion, that is, hemodilution concerns [14]. In open wound femur aspiration during THA or TKA, bone marrow sample can be aspirated without this concern since the suction needle is less likely to puncture any other tissue except bone marrow itself and also there is only a small force applied on suction needle for gliding along the bone marrow cavity as compared to the force applied in bone puncturing method. Above all, the suction needle end is not beveled.

It was very interesting that Narbona-Carceles and colleagues [14] were able to filter bone marrow through 100 *μ*m filters. On the other hand, we diluted the bone marrow with equal volume of 2 mM EDTA and then filtered it through a sieve of pore size 1.25 × 1.25 mm^2^. Given the much larger pore size we used, filtration of bone marrow was still slow. That indicated that the bone marrow in our aspirations was more viscous than the bone marrow aspirated by bone puncturing method [14]. Age could be a factor for this observation. Narbona-Carceles and colleagues [14] used aspirations from the patients in the age range of 64–75 years. On contrary, we used much younger patients in the age group of 51–64 years.

Narbona-Carceles and colleagues [14] aspirated 5 mL bone marrow from iliac crest, distal femur, and proximal tibia. In our procedure, the average volume aspirated from distal femur (during TKA procedure) was 4.87 ± 0.63 mL; additional gliding of suction needle in marrow cavity did not increase the aspirated volume (Figure 1(g)). On the other hand, in proximal femur (during THA procedure), the aspirated volume was 11.17 ± 1.15 mL which was 2.29 times higher than the distal femur. Narbona-Carceles and colleagues [14] reported recovery of MNCs per mL bone marrow as 0.67 ± 1.1 × 10^6^ in distal femur aspirate and 1.70 ± 4.8 × 10^6^ in proximal tibia aspirates. On the other hand, we showed the MNCs per mL bone marrow as 11.74 ± 1.21 × 10^6^ and 12.37 ± 2.28 × 10^6^ from proximal femur and distal femur, respectively (Figure 1(f)). MNCs number was 18.46-fold higher in our procedure in distal femur group than that in bone punctured procedure [14]. This could be due to the reason that we conducted investigation in much younger patients. Narbona-Carceles and colleagues [14] reported recovery of MSCs (per mL original bone marrow) as 2.90 ± 6.6 × 10^5^ in distal femur aspirates and 3.25 ± 2.6 × 10^5^ in proximal tibia aspirates on days 40.1 and 40.7, respectively. On the other hand, we showed the viable MSCs (per mL original bone marrow) in proximal and distal femur as 4.10 ± 0.72 × 10^6^ and 2.56 ± 0.69 × 10^6^ (Figure 1(f)) on days 18.39 ± 0.41 and 18.7 ± 1.13, respectively. Hence, comparing with bone punctured procedure for distal femur [14], our procedure provided 8.82-fold higher number of viable MSCs and that was on day 18.7 ± 1.13 as compared to bone punctured method on day 40.1 [14].

In brief, we presented a simplified procedure for bone marrow aspiration that is novel, less cumbersome, and more ethical towards patients undergoing joint replacement. By comparing distal femur aspirations, we obtained 18.46-fold higher number of viable MNCs per mL bone marrow than that obtained by Narbona-Carceles and colleagues [14]. By comparing distal femur aspirations, we obtained 8.82-fold higher number of viable MSCs per mL bone marrow than that obtained by Narbona-Carceles and colleagues [14] and at much earlier day. Based on our data, we strongly recommend proximal femur of THA patients as the preferred site for bone marrow collection for MSCs isolation simply because more volume can be aspirated from this site as compared to distal femur of TKA patients.

### 4.2. Micromass Chondrogenesis and Assessment

We generated micromass cartilage (also referred to as bioengineered cartilage like material) from the MSCs we isolated from bone marrow and characterized it (Figures 5–10). In the bioengineered micromass cartilages we generated (Figure 5(c)), we showed that collagen was present at week 1 to 4 as determined by Masson's trichrome staining (Figure 6(a)). Higher magnification (lower panels, Figure 6(a)) showed that collagen (blue colored fibers) increased from week 1 through week 4 indicating that cells secreted extracellular collagen in an increasing manner as micromass cartilage grew in size along with cell size (Figures 5(c) and 6(a)). This showed that chondrocytes are highly metabolic in function. This was supported by TEM studies of micromass cartilage chondrocytes at week 4 in which we showed that chondrocytes were metabolically active (Figure 10). Other groups have shown that human bone marrow MSCs-derived chondrogenic pellets, at day 14, showed the presence of collagen determined by Masson's trichrome staining [2]. Micromass cartilage showed the presence of proteoglycans as determined by safranin O staining, at low level at weeks 1 and 2 but prominently at weeks 3 and 4 (Figure 6(b)) similar to staining in native articular cartilage (Figure 6(b)). Pellet cartilage showed the presence of proteoglycans at week 2 determined by safranin O staining [2]. Zhang lab showed the presence of proteoglycans in micromass cartilage at weeks 1–3 as determined by toluidine blue staining [17].

Zhang lab showed the expression of COL II in micromass cartilage at week 2 [17]. We reported that COL II began to be expressed at week 2 and became prominent afterwards in micromass cartilage (Figure 7(a)). Tuan laboratory showed that COL2A1 gene was expressed at day 14 and increased at days 21 and 28, the similar pattern of COL II protein expression we showed in micromass cartilages by IHC (Figure 7(a)). In human bone marrow MSCs-derived chondrogenic pellets, COL II was expressed at day 14 shown by IHC [2]. We showed that micromass cartilages express COL I at week 1 through week 4 though it has less intensity than COL II (week 3 not shown, Figure 7(b)). By using RTPCR technique, Tuan laboratory showed the expression of COL1A1 gene at days 7–28 in chondrogenic pellets [18]. Chondrogenic cultures at weeks 1, 2, and 3 presented COL I expression [19]. COL I is not present in native articular cartilage and is present in bone as shown in our positive control (Figure 7(b)). Why is COL I expressed in* in vitro* chondrogenesis? There are two possibilities: (i) it is difficult to mimic chondrogenesis conditions (pH, oxygen tension, and autocrine factors) of native cartilage with those of in* in vitro* chondrogenesis and some cells can be reprogrammed to produce COL I [20–22]; (ii) during development, there is a cellular condensation event prior to joint development that has been shown to be rich in type I collagen [23].

Expression of cartilage hypertrophy markers by MSCs undergoing chondrogenesis raises concern for tissue engineering application for MSCs, because hypertrophy would result in apoptosis and ossification [18]. COL X is expressed at low level at day 7 and day 14 but increased at days 21 and 28 in pellet culture [18]. Besides low level of expression of COL X at weeks 3 and 4, we did not see any apoptosis in our micromass cartilages (Figures 8(a) and 8(b)). Apoptosis was present in pellet culture but not in micromass culture at week 2 as shown by IHC and TEM [17]. We showed that there was no apoptosis in micromass cartilages at any time point (Figure 8(b)) and also we did not see any apoptotic bodies in chondrocytes by TEM studies at week 4 in micromass cartilage (Figure 10). We used involuting mammary gland as control since it showed apoptotic epithelial cells for comparison (Figure 8(b)).

Aggrecan is the major proteoglycan in the articular cartilage. It is important in the proper functioning of articular cartilage because it provides a hydrated gel structure that endows the cartilage with load-bearing properties. Cartilage contains up to 10% proteoglycan consisting of mainly the large aggregating chondroitin sulfate proteoglycan aggrecan. Aggrecan and collagen II are the major proteins of cartilage [24]. Micromass cartilages showed aggrecan expression at all the time points (Figure 9(a)). Aggrecan expression has been shown in pellet and micromass culture by other investigators [17]. Eyre reported that COL VI accounts for less than 2% of total collagen in bovine cartilage [25, 26]. In articular cartilage, COL VI is found and is maintained at low levels, forming a microfibrillar network once secreted by the cell [27]. COL VI is expressed in normal cartilage and increases in OA cartilage [28, 29]. Micromass cartilage showed COL VI expression (Figure 9(b)). Knockdown of COL VI affected gene expression of aggrecan, biglycan, and SOX9 during* in vitro* chondrogenesis. COL VI was shown to be important in resisting applied strains [30].

Transmission electron microscopy images of micromass cartilage from the upper middle area at week 4 are shown (Figures 10(a)–10(e)) which indicated that chondrocytes are normal and are metabolic in nature. Zhang lab showed well-developed Golgi in chondrocytes and collagen fibers in extracellular matrix in week 3's chondrogenic cultures [17]. Similarly, we showed well-developed Golgi and collagen fibers in micromass cartilage (Figures 10(b) and 10(c)). Ichinose lab showed well-developed rER and abundant collagen fibers in day-14 pellet and well-developed rER and moderate collagen fibers in micromass cartilages [31]. Extensive rER is present in week-4 generated micromass chondrocytes (Figure 10(b)). Ultrastructure detail of micromass cartilage shows many similarities to the ultrastructure of human articular cartilage [32, 33]. Chondrocyte from human femur articular cartilage shows vacuoles, glycogen deposits, and rER similar to the structures we observed in the chondrocyte of micromass cartilage [33]. Roy and Meachim noted glycogen deposits, many mitochondria, and extensive rER in human nonfibrillated femur articular cartilage [32]. Lipid droplets are present in* in vitro* chondrogenesis from MSCs or chondrocytes [34, 35]. We observed lipid droplets in our micromass cartilages at week 4 (Figures 10(a), 10(c), and 10(d)). Presence of lipid droplets is not considered a sign of degeneration since lipid droplets are present in normal cartilage though they increase with aging [36, 37] and the exact role of lipid droplets is not yet clear. TEM of chondrocytes derived from infrapatellar fat derived stem cells showed a number of lipid droplets [35].

## 5. Conclusion

A simplified method for the aspiration of bone marrow was established from the femur of patients undergoing TKA or THA. The method provides an economical source of MSCs. A simplified procedure was described to isolate MSCs from the aspirated bone marrow. MSCs were characterized including extensive characterization through chondrogenesis. The detailed SOPs we provided will help researchers and clinicians.

## Figures and Tables

**Table 1:** Reconstitution of incomplete chondrogenesis medium.

Reagent	Source	Stock solution (storage)	Final conc.	Mix and filter sterilize
DMEM with high glucose, 1x	D5671 (Sigma-Aldrich)		1x	475 mL

L-Ascorbic acid 2-phosphate trisodium salt	323-44822 (Wako)	5 mg/mL in dH_2_O (4°C)	50 *μ*g/mL	5 mL

L-Proline	P5607 (Sigma-Aldrich)	50 mg/mL in dH_2_O (4°C)	50 *μ*g/mL	500 *μ*L

Insulin-transferrin-selenium-sodium pyruvate (ITS-A) (100x)	51300-044 (Gibco by Life Technologies)		1x	5 mL

Sodium pyruvate (100 mM)	11360-070 (Gibco by Life Technologies)		1 mM	5 mL

Penicillin-streptomycin (10,000 U/mL)	15140-122 (Gibco by Life Technologies)		100 U/mL	5 mL

GlutaMAX supplement (100x)	35050-061 (Gibco by Life Technologies)		1x	5 mL

Linoleic acid (1 gm/mL)	L1012 (Sigma-Aldrich)	100 *μ*g/*μ*L [dilute 5 *μ*L (1 gm/mL) to 50 *μ*L in dH_2_O]	5.33 *μ*g/mL	26.7 *μ*L

Dexamethasone (MW: 392.46)	D4902, (Sigma-Aldrich)	*Solution-A* (10 mM) (dissolve 25 mg in 6.37 mL EtOH and store at −20°C) *Solution-B* (1 mM): dilute 100 *μ*L solution-A to 1 mL in dH_2_O	100 nM	50 *μ*L (solution-B)

**Table 2:** List of antibodies used for IHC of micromass cartilages.

	Primary antibody	Secondary antibody
Type II collagen (COL II)	Mouse anti-COL II, monoclonal, II-II6B3, DSHB	Biotinylated horse anti-mouse IgG, rat adsorbed, BA-2001, Vector

Type X collagen (COL X)	Mouse anti-COL X, monoclonal, 2031501001, Quartett, GE	The same as above

Type I collagen (COL I)	Mouse anti-COL II, C2456, Sigma-Aldrich, ascites fluid	The same as above

Aggrecan	Rabbit polyclonal to aggrecan, ab36861, abcam	Biotinylated horse anti-rabbit IgG, BA-1100, Vector

Type VI collagen (COL VI)	Rabbit polyclonal to COL VI (Biotin), ab6583, abcam	None

(i) Primary and secondary antibodies were diluted in blocking buffer.

(ii) Negative control was without primary antibody and with mouse IgG (for types II, I, and X) and with rabbit IgG, polyclonal (ab27478, abcam), for aggrecan and COL VI.

(iii) For antigen retrieval for COLs I, II, X and aggrecan, the hydrated tissue sections were treated with pepsin (P-7000, Sigma-Aldrich; 4 mg/mL in 0.01 N HCl) for 10 min at 37°C, washed with dH_2_O (4x, 1 min), and then treated with hyaluronidase (H-3506, Sigma-Aldrich) solution at 1 mg/mL (in 0.1 M phosphate buffer, pH 5.0) for 30 min at 37°C and washed with dH_2_O (4x, 1 min).

(iv) For antigen retrieval for COL VI, hydrated sections were treated with 20 *μ*g/mL proteinase K (EO0491, Thermo Fisher) for 15 min at 37°C.

(v) Blocking buffer constituted of PBS-T containing 2% BSA (ALB-001, albumin-bovine serum fraction V, Bioshop) and 2% horse serum (16050122, GIBCO).

(vi) PBS-T was composed of D-PBS containing 0.05% Tween-20 (TWN510, Bioshop).
